# ME/CFS and the emotional toll of persistent disbelief: from epistemic to affective injustice

**DOI:** 10.1007/s11019-026-10388-6

**Published:** 2026-08-14

**Authors:** Sven Walter

**Affiliations:** Institute of Cognitive Science, Osnabrück University, Wachsbleiche 27, D-49069 Osnabrück, Germany

**Keywords:** Affective injustice, Epistemic injustice, Marginalization in healthcare, ME/CFS, Medical gaslighting, Philosophy of medicine

## Abstract

*Myalgic Encephalomyelitis/Chronic Fatigue Syndrome* (ME/CFS) is a debilitating chronic illness whose sufferers are frequently met with disbelief, stigmatization, and psychologization in both clinical and social contexts. Recent work has used the concept of “epistemic injustice” to illuminate important dimensions of this problem, especially the ways in which ME/CFS patients are discredited as knowers and denied adequate interpretive resources. This paper argues that the harms associated with persistent disbelief in ME/CFS are not exhausted by this epistemic dimension. Drawing on recent philosophical work on *affective injustice*, I argue that persistent disbelief can also impair patients’ affective lives in distinctive and socially patterned ways. More specifically, I argue that individuals with ME/CFS are burdened by unjust affective expectations, pressured into norm-conforming forms of emotional self-presentation, exposed to pathologizing and gaslighting interpretations of their affective experience, denied uptake for apt emotional responses, and exploited through forms of emotional labor and affective appropriation. Conceptualizing these harms in terms of affective injustice, I argue, helps to capture dimensions of marginalization that a focus on testimony and understanding alone leaves obscure. The paper concludes by considering objections and drawing out implications for healthcare and philosophy of medicine.

## ME/CFS: history, models, and burdens

*Myalgic Encephalomyelitis/Chronic Fatigue Syndrome* (ME/CFS) is a debilitating chronic multisystem illness that affects a substantial minority of the population across all ages, races, and socioeconomic groups (e.g., Valdez et al. 2018). Prevalence estimates range from 0.2% to 1% (e.g., Lim et al. 2020). Women are affected about two to three times more often (e.g., Thomas et al. 2022). The etiology and pathophysiology of ME/CFS remain unclear, and there are no definitive biomarkers (e.g., Clarke et al. 2025). Its diagnostic symptomatology includes profound fatigue, post-exertional malaise, noise and light sensitivity, unrefreshing sleep, cognitive dysfunction (“brain fog”), pain, orthostatic intolerance, and immune disturbances (e.g., Lu et al. 2025). Mildly affected patients are able to work at a reduced level, though often only at the cost of giving up most leisure and social activities; moderate cases are housebound, experiencing substantial restrictions in activities of daily living; severe cases remain bedridden and dependent on external care for extended periods. Understandably, ME/CFS causes not only hefty personal suffering, but also considerable economic costs, including healthcare expenditures and losses in productivity. Recent estimates placed its annual burden at $36–51 billion in the United States (Jason and Mirin 2021) and €40 billion in Europe (Pheby et al. 2020). But even these numbers might be grotesquely optimistic or, at the very least, outdated. In a recent state-of-the-art damage assessment, the modelled annual costs attributable to ME/CFS rose from €20.9 billion in 2020 to €30.9 billion in 2024 *in Germany alone* (Daniell et al. 2025).

What is now commonly called ME/CFS was officially classified by the World Health Organization as a neurological disorder in 1969. It has been associated with a suspected viral outbreak at London’s Royal Free Hospital in 1955 that affected almost 300 nurses and other staff members (The Medical Staff of the Royal Free Hospital 1957). Following that, “benign myalgic encephalomyelitis” came to be recognized as a clinical syndrome (Acheson 1959). In 1970, however, two (male) British psychiatrists recast the Royal Free outbreak as a case of “epidemic hysteria.” They explicitly cited “the high attack rate in females” as support for what they took to be “a much more likely” (McEvedy and Beard 1970, p. 7) psychogenic nature of the condition, eventually referring to those affected as “pathological hysterics” (McEvedy and Beard 1973, p. 149). Their brazen reinterpretation fitted neatly into a broader psychogenic framing of the condition that continued to shape medical, public, and social-policy discourse for decades (Hunt 2025). Within this broader climate, the formally neutral label of a “medically unexplained condition” was misappropriated to cast individuals with ME/CFS as hypochondriacs whose psychosomatic symptoms were perpetuated or maintained by their own dysfunctional illness beliefs and avoidance behaviors (e.g., Deary et al. 2007). ME/CFS was stigmatized as the “Yuppie Flu” and socially branded as a fashionable neurasthenic malaise of affluent good-for-nothings who sought medical legitimacy for their inability, or unwillingness, to cope with modern life (e.g., Wessely 1990). Leading researchers suggested that patients needed a “simple mental state examination” (Harvey and Wessely 2009, p. 2) more than “real” medical care and scientific investigation—or, for that matter, access to welfare support and income-protection benefits (Hunt 2025).

This old opposition survives today in the fault line between “biopsychosocial” and “biomedical” models of ME/CFS. On the one hand, parts of the medical establishment still regard it as a condition that, while potentially virally triggered, may be prolonged and exaggerated by interacting cognitive, behavioral, and social factors (e.g., Fan et al. 2026; Kuut et al. 2024), including patients’ coping styles (e.g., Creswell and Chalder 2001), illness perceptions (e.g., Edwards et al. 2001), and personality traits, for instance perfectionism (e.g., Deary and Chalder 2010) or neuroticism (e.g., Sáez-Francàs et al. 2014). At its most extreme, persistent somatic symptoms have been attributed to patients’ prior expectations (e.g., Joffe and Elliott 2023) and their mistrust of scientific authorities (e.g., Matta et al. 2023) or even, more generally, of the state (e.g., Zipper et al. 2024). On the other hand, in line with patient organizations who have been especially active in contesting a biopsychosocial framing, a biomedical model is increasingly corroborated (e.g., Thoma et al. 2024). Current biomedical research documents immunological (e.g., Jonsjö et al. 2020), neurological (e.g., Komaroff and Dantzer 2025), metabolic (e.g., Scheibenbogen and Wirth 2025), and vascular (e.g., Nunes et al. 2026) abnormalities as factors in ME/CFS and has begun to identify genetic susceptibility factors (DecodeME Collaboration 2025).

Whatever its precise causes and nature, though, ME/CFS is associated with a range of often devastating hardships. Individuals with ME/CFS face an increased risk of suicidal ideation and suicide attempts (e.g., König et al. 2024; McManimen et al. 2018), often live under conditions of marked economic precariousness (e.g., Castro-Marrero et al. 2019), and show elevated rates of overlapping chronic pain conditions, including migraine, fibromyalgia, low back pain, and irritable bowel syndrome (e.g., Fall et al. 2024). Some also develop malnutrition because nausea, food intolerance, sensory hypersensitivity, or difficulties chewing and swallowing severely restrict nutritional intake (e.g., Baxter et al. 2021), with potentially fatal consequences (Archer 2024).

In addition to such more measurable burdens, ME/CFS also affects patients’ lived experience, often in ways that are no less damaging. ME/CFS impinges on the overall quality of life more severely than a number of other severe illnesses, including cancer (e.g., Hvidberg et al. 2015) and multiple sclerosis (e.g., Kingdon et al. 2018). One reason for this is that individuals with ME/CFS are burdened not only by their condition itself, but also by *persistent disbelief*. Medical professionals, the healthcare industry, the media, and sometimes even significant others regularly trivialize the symptoms or question the legitimacy of ME/CFS, resulting in marginalization, stigmatization, and, thereby, additional suffering (e.g., König et al. 2024; Sirotiak and Thomas 2025; Nezamdoust and Ruel 2026; Ma et al. 2025). Drawing on a notion coined by Fricker (2007), recent attempts to explicate the various kinds of wrongs in the medical and social treatment of ME/CFS have come to conceptualize this further layer of harm as a form of “epistemic injustice” that impairs patients’ ability to participate in shared practices of knowledge acquisition and distribution (see Sect. “ME/CFS and epistemic injustice”).

While I agree with the general thrust of the epistemic injustice framework, it does not, I think, exhaust what needs to be said to understand the subjective toll of persistent disbelief, which cannot merely undermine patients’ capacity to access, produce, and validate *knowledge*. It can also impair their affective attunement to themselves and the world, their meaningful connectedness to others, and even their sense of autonomy, authenticity, and agency, causing distinctively *affective* harm. In this paper, I suggest that such harm may be understood as a manifestation of “affective injustice” (e.g., Srinivasan 2018; Whitney 2018). My aim in doing so is twofold. I want to add to philosophical discussions of affective injustice by bringing into view an instance that has thus far remained unexamined, and I want to enrich interdisciplinary work on the marginalization of individuals with ME/CFS by offering a philosophical conceptualization of its distinctively affective dimension.

The remainder of this paper proceeds as follows. Section “ME/CFS and epistemic injustice” sets the stage by briefly canvassing recent applications of the concept of epistemic injustice to ME/CFS, acknowledging the explanatory power of the framework, but also some problems and limitations. Section “From epistemic to affective injustice” then introduces the corresponding notion of affective injustice. Based on this, Section “ME/CFS and affective injustice” argues that individuals with ME/CFS experience various affective harms that are structurally analogous to those often identified as forms of affective injustice. Section “Conclusion: objections and implications” concludes by addressing some objections and drawing out some broader implications.

## ME/CFS and epistemic injustice

In her groundbreaking work, Fricker argued that unjust social power dynamics can undermine individuals’ fundamental ability to participate in epistemic practices of knowing, understanding, and being understood. The harm someone experiences when they are mistreated specifically “in their capacity as knowers” (2007, p. 20), Fricker argued, constitutes a distinct kind of *epistemic injustice*. Such injustice arises when individuals are curtailed in their ability to contribute to or participate in shared practices of inquiry, interpretation, and understanding, not on legitimate epistemic grounds, but because identity-based social prejudices systematically disadvantage them. Fricker’s work has been immensely important in helping us see how objectionable social practices can inflict distinctive harms on marginalized individuals by systematically impeding their epistemic standing. One particularly important domain in which this has recently proved fruitful is healthcare (e.g., Carel and Kidd 2014), including, specifically, efforts to make sense of the harm experienced by individuals with ME/CFS.

Blease et al. (2017) argue that individuals with ME/CFS are especially vulnerable to Fricker’s two central forms of epistemic injustice: testimonial and hermeneutical injustice. On their view, a testimonial form of epistemic injustice arises when unfamiliarity with or disbelief in ME/CFS leads clinicians not to take symptom reports seriously, hesitate to apply the diagnosis, or question the value of specialist referral. Especially when combined with negative stereotyping of patients as burdensome, unreliable, or difficult (see Sect. “Unjust expectations and affective policing”), such uncertainty can translate into a deflation of their credibility, with the result that their testimony is not acted upon as it otherwise might be. A hermeneutical form of epistemic injustice, Blease et al. (2017) hold, arises when patients are denied appropriate interpretive resources to make sense of their symptoms, for instance because standard medical training leaves clinicians unable to describe those symptoms as cohering into a legitimate condition, or because a primarily biopsychosocial framework disregards or overrides patients’ own accounts of their suffering. The consequence is not merely disagreement between doctors and patients, but a structurally biased exchange in which the latter are less readily believed, less adequately understood, and less able to make sense of themselves. In line with this, Hunt et al. (2024) describe that patients often report that they are treated as attention-seeking, dramatic, or malingering, that their symptoms are “psychologized,” and that serious physical complaints are often not investigated, resulting in a constant struggle for “epistemic-existential validation” (ibid., 1133) in their negotiation of “socio-epistemic hierarchies” (ibid., 1135). Yet, Hunt et al. (2024) explicitly extend the notion of epistemic injustice by linking it to threats to identity and personhood, chronic stress, and the wider life-world consequences of being “de-centred in patient-centred care” (ibid., 1133). Relatedly, Runacres et al. (2025) argue that the concept of epistemic injustice helps illuminate not only how ME/CFS burdens patients as knowers, but also how it can affect their sense of self and their struggle to manage social acceptability and render their illness intelligible. Lastly, de Boer (2021) and de Boer and Slatman (2023) use the notion of epistemic injustice as a conceptual lens to show how media discourse can affect the credibility of individuals with ME/CFS.

This line of research is immensely important. It can tell us a lot about the hurdles individuals with ME/CFS face, allowing for a richer understanding of what it is like to navigate healthcare, policy, and everyday life in a world that doubts one’s condition and testimony and limits one’s means to make sense of one’s own experience. However, it is not without problems and leaves important issues underaddressed.

First, parts of the literature give the impression that the main source of epistemic injustice is the biopsychosocial framing which treats ME/CFS as largely “in the head” rather than a “real disease,” setting up an opposition between clinicians and patients that, given prevailing social hierarchies, the former are structurally positioned to win. However, it would be a mistake to assume that a biomedical model would automatically avoid all epistemic injustices. As de Boer (2021) has pointed out, it may enhance patients’ credibility, but it may also sustain reductive ideas about what illness is and whose knowledge counts as authoritative. It may therefore not so much free patients from epistemic injustice as move them from one precarious position to another. Testimonial and hermeneutical injustice may persist even under a biomedical model: patients’ accounts may be subordinated to test results and clinical authority and the subjective and existential dimensions of their illness may remain marginalized.

Second, some applications of the notion of epistemic injustice seem to be more liberal than Fricker intended, treating a wide range of harms—stigma, delegitimization, poor communication, and threats to one’s identity—as though they were all forms of epistemic injustice. But once the framework is made to cover so much, it may seem as if it no longer marks out a distinctive kind of epistemic injustice, but functions primarily as a rhetorically vivid umbrella term for all sorts of harm associated with living with a contested illness. Even with regard to the properly epistemic, the problem sometimes seems to be overstated because potentially well-motivated clinical uncertainties are too readily castigated as identity-based prejudices (Byrne 2020). Given the heterogeneity of ME/CFS, the absence of definitive biomarkers, and the ongoing uncertainty regarding its causes and mechanisms, diagnostic hesitation or scrutiny of symptom reports may reflect legitimate clinical caution rather than a testimonial injustice by the medical establishment.

Third, and most importantly for current purposes, parts of the literature on epistemic injustice already acknowledge that the epistemic dimension is actually not the whole problem. Hunt et al. (2024), for instance, frame the issue in terms of experiences of “existential erasure” (ibid., 1134), with patients describing the experience of not “being treated as a human being” (ibid.). Similarly, Runacres et al. (2025) stress that patients become entangled in “stigma and perceptions of burden” (ibid., 14) and experience themselves as subjected to “the emotional toll of being wrongfully accused” (ibid.). Accordingly, even if the epistemic injustices generated by disbelief about ME/CFS were fully resolved, their social and clinical treatment could still burden patients in ways that are potentially unjust. What is at issue is, therefore, not merely patients’ standing as *knowers*, but also the normative standing of their *affective life*. The natural next step is to make this dimension more explicit.

## From epistemic to affective injustice

Following Fricker’s (2007) work on epistemic injustice, others have argued that asymmetries in social power can not only wrong marginalized individuals in their capacity as knowers, but can also systematically curtail their *affective lives*. When morally problematic dominant norms, ideologies, attitudes, evaluative dispositions, and social practices systematically impair the ability of individuals with less social power to express, regulate, and share their emotions, moods, or feelings, they have been said to be wronged in their “capacity as an affective being” (Archer and Mills 2019, p. 76). Since Srinivasan (2018) and Whitney (2018) independently dubbed this kind of wrong “affective injustice,” the literature is burgeoning.

A first wave of philosophical work on affective injustice focused on more general manifestations of oppression, demonstrating how social power constrains not only what marginalized individuals can know or understand, but also what affective responses they can feel and are entitled to express, and whether those responses are recognized as intelligible or warranted (Srinivasan 2018; Whitney 2018). More recently, this idea has, again, been applied to the healthcare context. Bogaert (2025), for instance, regards norms and practices that minimize, hide, or silence the affective experiences of patients, families, and healthcare professionals as manifestations of affective injustice. Related work examines the dismissal and pathologization of patients’ affective responses in chronic pain, borderline personality disorder, and psychiatric contexts (Lavallée and Gagné-Julien 2024; Pismenny 2026; Oredsson and Lippert-Rasmussen 2025), as well as the way unjust social practices can distort patients’ feelings and lead therapeutic interventions to exacerbate existential distress (Puddifoot 2025; Vespermann and Tirkkonen 2025).

These examples illustrate how fruitful the notion of affective injustice might be for the healthcare domain. At the same time, however, they also bring to the fore a problem already indicated in Sect. “ME/CFS and epistemic injustice”: like “epistemic injustice,” the label “affective injustice” seems to be applied to a quite heterogeneous range of phenomena. This can seem to lead to a loss of analytical sharpness that renders it unclear what, exactly, unifies these phenomena as a distinctive kind of genuine *injustice* (Walter 2026). Accordingly, before the notion of affective injustice is applied to the harms experienced by ME/CFS patients, it is helpful to briefly clarify both how this worry might be avoided and how, more generally, the notion will be understood in what follows.

Elsewhere, I have argued for an *ameliorative* approach to affective injustice (Walter 2026). The basic idea is that instead of appealing to some antecedent, independently established meaning that allows us to capture what we have in mind, we should justify the notion of affective injustice by demonstrating that and how it can serve certain legitimate theoretical and practical goals (e.g., Haslanger 2012). That is, we should deliberately *engineer* the notion to help us track, interpret, evaluate, and contest a range of morally and politically important phenomena that are not grouped together by any established linguistic practice, but are unified by our descriptive, normative, and pragmatic aims that make them stand out as a distinctive class of power-laden affective wrongs.^1^ This is, in a nutshell, the background against which Sect. “ME/CFS and affective injustice” will now apply the notion of affective injustice to ME/CFS. I will distinguish five “stages” of social interactions at which marginalized individuals can be affectively harmed in ways that have been regarded as injustices. What justifies this, on the present view, is precisely that they are the phenomena the ameliorative account just outlined is designed to identify, interpret, and remedy. And as I will argue, individuals with ME/CFS are burdened, in some form or other, with each of them.

## ME/CFS and affective injustice

Since the philosophical literature on affective injustice has come to cover an increasingly variegated range of phenomena, a more fine-grained taxonomy can be helpful if we want to understand precisely how people’s affective lives can be systematically wronged. I suggest organizing the terrain by focusing on five “stages” of social interactions.^2^ In such interactions, others can (1) *expect* us to feel in a certain way, (2) attempt to *elicit* certain feelings in us, (3) *interpret* our feelings, (4) *respond* to our feelings, and (5) *benefit* from them. At each of these stages, power asymmetries can cause affective harm that, in light of Section “From epistemic to affective injustice”, may qualify as a manifestation of affective injustice. This section draws on this fivefold structure to bring into view related but distinctive kinds of such injustice in the context of ME/CFS. Table 1 summarizes the five stages, the corresponding forms and subforms of affective injustice distinguished below, and their application to ME/CFS.^3^

**Table 1:** Five stages, forms, and subforms of affective injustice and their application to ME/CFS

Stages	Forms	Subforms	Application to ME/CFS
Unjust expectations	*Affective policing*: dominant emotional regimes pre-structure which feelings certain individuals are expected to express or suppress.	*Affective imperialism*: emotional regimes of dominant cultures are imposed on subjugated groups. *Affective stereotyping*: certain individuals are expected to feel or express emotions in stereotyped ways. *Affective smothering*: individuals suppress apt feelings in anticipation of dismissal, pathologization, or sanction.	*Affective imperialism*: responses to ME/CFS are shaped by affective regimes of sanism and neoliberal ableism rooted in longer colonial regimes of normalcy and productivity. *Affective stereotyping*: patients are stereotyped as difficult, attention-seeking, hysterical, malingering, or uncooperative. *Affective smothering*: patients actively suppress anger, fear, or grief to avoid dismissal, pathologization, or loss of support.
Unjust elicitations	*Affective extraction*: others actively try to induce particular feelings, experiential attitudes, or affective performances in certain individuals.	*Affective solicitation*: others exert informal pressure on certain individuals to display norm-conforming feelings. *Affective coercion*: support or credibility are made conditional on displaying norm-conforming feelings. *Affective intimidation*: certain individuals are pushed into general feelings of self-doubt, insecurity, or helplessness.	*Affective solicitation*: patients are urged to think positive, push through, remain hopeful, or show willingness. *Affective coercion*: credibility, care, or benefits are made conditional on appearing open, compliant, and recovery-oriented. *Affective intimidation*: repeated disbelief induces feelings of self-doubt or helplessness.
Unjust interpretation	*Affective distortion*: others systematically misunderstand, misread, or misconstrue what certain individuals feel.	*Affective pathologizing*: apt feelings of certain individuals are recoded as symptoms of disorder. *Affective gaslighting*: certain individuals are led to doubt the aptness or reality of their own feelings. *Affective inarticulation*: certain individuals are deprived of confidence in or resources for making sense of their own affective life.	*Affective pathologizing*: anger, grief, fear, or frustration are recoded as anxiety, catastrophizing, or dysfunctional beliefs. *Affective gaslighting*: persistent disbeliefs make patients doubt whether their symptoms or feelings are real or indeed “all in the head.” *Affective inarticulation*: persistent disbelief makes it harder for patients to name, trust, or explain their affective life.
Unjust responses	*Affective dismissal*: others react to properly understood feelings by refusing to acknowledge their significance.	*Affective invalidation*: apt feelings of certain individuals are dismissed as exaggerated, unfounded, or inappropriate. *Affective silencing*: apt feelings of certain individuals are actively prevented from receiving uptake or public recognition.	*Affective invalidation*: patients’ suffering is minimized as exaggeration, stress, or overreaction, while apt anger is cast as militancy. *Affective silencing*: public ridicule or “militant patient” stereotypes block uptake of patients’ anger and outrage.
Unjust benefits	*Affective exploitation*: others take advantage of the emotional labor certain individuals must perform under conditions of social asymmetry.	*Affective double burdening*: certain individuals must manage others’ needs or reactions in response to their own suffering. *Affective appropriation*: the affective lives of certain individuals are used for the purposes of dominant actors or institutions.	*Affective double burdening*: patients must educate others, reassure relatives, and appear credible while already exhausted. *Affective appropriation*: media, stock images, or illness entrepreneurs exploit simplified or hopeful narratives of ME/CFS.

### Unjust expectations and affective policing

Our affective lives are shaped not only by what others do in concrete social interactions, but already by how their *affective expectations* pre-structure any such interaction. Accordingly, even before a single word is exchanged, marginalized individuals can be affectively wronged by the socially entrenched scripts and evaluative habits of dominant groups that operate, explicitly or implicitly, to *police* what “people like them” are supposed to feel and which feelings “people like them” are expected to express or suppress. Three forms of such *affective policing* (e.g., Pismenny 2026) are *affective imperialism* (e.g., Archer and Matheson 2022, 2023), *affective stereotyping* (Cherry 2023), and *affective smothering* (Bailey 2018). *Affective imperialism* occurs when dominant cultures impose their affective norms on subordinated cultures while castigating the latter’s affective styles as inappropriate or deviant. *Affective stereotyping* is a kind of “imperialism turned inward” through which dominant groups within a society impose expectations regarding how less powerful groups are to feel. *Affective smothering* occurs when marginalized individuals pre-emptively suppress potentially apt feelings because they can reasonably anticipate that they will be denied uptake, pathologized, or otherwise sanctioned.

The arguably most prominent manifestation of affective policing in the context of ME/CFS is *affective stereotyping*. It has been amply documented that individuals with ME/CFS are burdened by stereotypes that police their affective lives in much the same way as more familiar oppressive stereotypes do in other domains, such as the expectation that boys don’t cry or that Black women are “angry.” Individuals with ME/CFS are often stigmatized as affectively and morally suspect in a distinctive way (e.g., Åsbring and Närvänen 2002; Dickson et al. 2007; Hunt et al. 2024), with serious negative consequences for their well-being (e.g., Baken et al. 2018). In part owing to the prevalence of a biopsychosocial model, they have been cast as “heartsink” patients “whose only common thread seems to be the distress they cause their doctor” (O’Dowd 1988, p. 528), or as “attention seeking” (Snell et al. 2024, p. 232) “hypochondriacs or malingerers” (Brown et al. 2017, p. 698) who are simply “making it up” (Tenhave et al. 2025, p. 8). Importantly, such stereotyping is markedly *gendered* (see also Sect. “Unjust interpretation and affective distortion”). Since women are more readily assimilated to the archetype of the “hysterical woman” with its entrenched scripts of emotional instability, fragility, and dependence, disbelief about ME/CFS as a “non-disease” is especially pronounced for them (e.g., Murray et al. 2019). By contrast, male patients are often portrayed as physically active and successful men who have been suddenly struck down by illness, which makes a biomedical explanation appear more plausible, suggesting that their complaints are more legitimate (e.g., de Boer and Slatman 2023).

Importantly, it is not only the overt deployment of stereotypes in particular encounters that is harmful. Their very *social availability* places individuals with ME/CFS in *affective double binds* in which whatever they do is liable to reinforce their marginalization. On the one hand, just as Black women who refuse to suppress their anger further reinforce the Angry Black Woman stereotype (e.g., Srinivasan 2018), individuals with ME/CFS who insist on the gravity of their condition or express anger at their treatment risk confirming stereotypes of being attention-seeking, difficult, or mentally unstable (e.g., Spandler and Allen 2017), or even escalating them into charges of being “fanatical” (de Boer and Slatman 2023, p. 602) or “militant” (Blease and Geraghty 2018). On the other hand, if they avoid such social sanctioning by suppressing their apt affective reactions, they are affectively smothered (see below) and risk not being heard at all or not appearing sufficiently ill, thereby reinforcing the stereotype that their condition is “all in the head” (Sirotiak and Thomas 2025, p. 17698).

The same pattern of self-discrediting pressure arises in connection with another stereotype. Society frees those who occupy the “sick role” (Parsons 1951) from certain social expectations, such as work and other responsibilities. In return, however, it expects them to be “good patients” who defer to medical authority without resistance. Importantly, the “good patient” stereotype includes not only the expectation that ME/CFS patients seek to get well, but also that they affectively orient themselves to recovery in the right way: they must “show willing” (Hunt and Blease 2026, p. 477), and be neither too angry nor too frightened, so that they do not organize their lives too visibly around managing their energy or withdrawal from productivity (e.g., Cheshire et al. 2021). The result is again an affective double bind. If they refuse to be “good patients,” they reinforce prejudices that cast them as “obstructive and uncooperative” (Hunt et al. 2024, p. 1137), “psychologically unstable” (Snell et al. 2024, p. 230), “hysterical and malingering” (Spandler and Allen 2017, p. 133), or even as “anti-science” (Hsu 2024; Zipper et al. 2024). If, by contrast, they try to conform to the expectations of the “good patient” ideal, they have to suppress precisely those affective responses that may be most fitting to their situation, resulting, again, in *affective smothering*.

While stereotyping can drive patients into pre-emptively suppressing apt affective responses, this is not the only route into *affective smothering*. They may come to proactively mute themselves not (merely) to avoid being cast as difficult, but (also) because they can foreseeably anticipate more tangible repercussions. As Hunt et al. (2024) document, many patients experience medical encounters as so tightly scripted by entrenched structures that they are effectively “stuck” (ibid., 1136) when they fail to fit established clinical expectations, making meaningful help less likely. As one patient recounts: “I had to put on a face and pretend to be well […] otherwise, the eyes would roll” (Dickson et al. 2008, p. 466). Relatedly, patients may be affectively smothered by repeated exposure to healthcare structures in which they experience “a pressure to […] act a certain way or say the right thing” (Waite and Elliot 2021, p. 165) because clinicians and medical assessors hold “too much power” (Hunt et al. 2024, p. 1136)—power that is “too big for [them] to be able to influence” (ibid.)—so that dissent again threatens to carry quite practical penalties.

*Affective imperialism* may seem the least applicable to ME/CFS, as it is originally understood as a matter of one culture imposing its own emotional regime on another—for example, when Portuguese colonial rule in Angola sought to cultivate admiration and gratitude toward the colonizers through enforced commemorative practices (e.g., Archer and Matheson 2023). Yet, on the one hand, the notion has recently also been applied to *intra*cultural marginalization, for instance to the imposition of neurotypical majority norms on autistic individuals whose own affective styles are marked as inferior (Goffin and Archer 2025). If that counts as affective imperialism, there is no reason why the imposition of clinical affective regimes that demand trust and confidence, while treating distrust and fear as deviant, should not also qualify. On the other hand, even in its narrower sense, affective imperialism may still illuminate some underlying causes of the suffering of ME/CFS patients. Two broader normative regimes that shape society’s responses to ME/CFS are *sanism* (e.g., Spencer et al. 2025) and *neoliberal ableism* (e.g. Hunt 2025). The cultural logic of *sanism* casts individuals with psychological illnesses as less reliable and less authoritative over their own experiences, while *neoliberal ableism* ties human worth to productivity, self-sufficiency, and bodily normalcy, stigmatizing those who cannot meet fast-paced life standards. In the context of ME/CFS, sanism is (partially) responsible for the pathologization of patients’ affective responses to social and political conditions as consequences of mental instability or irrationality (e.g., Lavallée and Gagné-Julien 2024; Spandler and Allen 2017; Spencer et al. 2025), while neoliberalism legitimizes the prioritization of market interests over compassion and citizens’ rights that positions patients as a socio-economic problem to be corrected and reintegrated into productivity (Hunt 2025) and expects them to “show willing” to prove “deserving” (e.g., Hunt et al. 2024; Runacres et al. 2025) and, eventually, “go back to work” (Hunt et al. 2024, p. 1137). However, these affective regimes are not historically innocent. Critical work in settler colonial studies has detailed how sanist norms result from the universalization of Western psychiatric reason, whereby non-dominant experiences were pathologized and alternative ways of knowing were displaced (e.g., Dwornik 2021; Mills 2017). Similarly, neoliberal ableist norms of “industrious” citizens have historically been imposed through disability frameworks and services shaped by white settler colonial formations (Puszka et al. 2022) and through the racialized hierarchies propagated by them (Strakosch 2015). In light of this, the expectations with which ME/CFS patients are currently burdened may be understood as not merely reflecting prevailing local stereotypes, but as downstream expressions of longer colonial regimes of normalcy and productivity that may constitute a kind of affective imperialism in the narrow sense.

### Unjust elicitations and affective extraction

Social interactions can become sites of injustice not just because they are pre-structured by unjust passive expectations, but also because they involve active attempts to *elicit* certain affective responses. *Unjust elicitations* seek to induce affective experiences in others in ways that compromise their affective autonomy by making them bend to dominant normative demands (e.g., Pismenny et al. 2024). Three forms of such *affective extraction* are *affective solicitation* (e.g., Cherry 2019), *affective coercion* and *affective intimidation*. *Affective solicitation* occurs when dominant groups feel entitled to elicit stereotypical affective responses in marginalized individuals, typically through informal social pressure rather than overt threats or sanctions, for instance when women are told to “smile” or “relax” (Cherry 2019). This turns into *affective coercion* when noncompliance is personally or institutionally penalized. *Affective intimidation* occurs when marginalized individuals are not pressured into specific norm-conforming feelings, but into more general affective conditions of doubt and insecurity that make them easier to handle, as when women are raised in the belief that they are “too emotional” (e.g., Whitney 2023).

In the context of ME/CFS, *affective solicitation* often takes the form of well-meant advice, especially outside explicit healthcare settings, that is shaped by traditional stereotypes about healthy living and health improvement and may strike outsiders as mere “common sense,” but often fails to do justice to the lived experience of ME/CFS patients (e.g., Risør and Lillevoll 2021). One important example is the attempt to solicit the feelings of therapeutic hope and recovery-oriented optimism expected from “good patients.” Such attempts can take the form of familiar injunctions to maintain an “optimistic outlook” (Jelbert et al. 2010, p. 280) and to “think positive” (Lian and Robson 2017, p. 8), the comment that they “don’t look sick” (Smyth and Blitshteyn 2025, p. 2), or banal advice such as “We all get tired sometimes—just get a proper night’s sleep and you’ll feel better!” (Habermann-Horstmeier and Horstmeier 2024, p. e27). Again, such stereotypical solicitations can be markedly *gendered*, for instance when male patients are encouraged to “act like a man” (Lian and Robson 2017, p. 8), while female patients are reminded to “think of their husbands and children” (e.g. Widerberg 2005). More harshly, patients are often told that they have to “just pull themselves together” (Lian and Robson 2017, p. 8) and “keep going and push through” (Strassheim et al. 2021, p. 11). While such demands may still qualify as “mere” affective solicitation, they can start to shade into coercion, depending on how insistently they are voiced and what costs are attached to noncompliance.

Once workplace, welfare, and clinical institutions begin to sanction noncompliance in an attempt to “normalize” patient behavior they perceive as deviant (Åsbring and Närvänen 2004), calls for optimism and perseverance take on a more coercive character by pressing patients into affective behavior even when it fails to reflect how they truly feel and, even worse, increases their suffering. Such *affective coercion* may occur, for instance, when individuals with ME/CFS who want to remain credible as cooperative “good patients” who “deserve” help are pressured into feigned feelings and postures of positivity because they can anticipate further support only if they appear “open,” “willing,” and able to take a “leap of faith” (e.g., Broughton et al. 2017).

*Affective intimidation* occurs when patients are not pressed into specific feelings, but affectively “terrorized” (Pismenny et al. 2024, p. 161) through protracted disbelief, questioning, and dismissal into more general states of self-doubt and helplessness. Individuals with ME/CFS often report that an invalidating environment of disbelief and distrust make them feel as if they were “faking ill” (Fisher and Crawley 2013, p. 563), a feeling that can, over time, make “them doubt themselves” (Cheston et al. 2025, p. 14), make them ask “themselves if they are really ‘only crazy’ after all” (Ware 1999, p. 313), make them “second guess whether [they are] up to it” (Tenhave et al. 2025, p. 8), and wonder whether “it’s all just in their head” and they “just have to try harder” (Habermann-Horstmeier and Horstmeier 2023, p. 21). As one patient vividly describes their desperation: “I feel like every day I am battling to prove myself—that I am really this sick, that I am working hard, how exhausted I feel, how difficult things are for me, in order to avoid their judgements. But it feels like standing on the beach with a bucket trying to stop the tide coming in. No matter how hard I try, I can’t help their comments and attitudes creeping in and making me question myself” (Hale et al. 2020, p. 51). This dynamic amounts to a form of “affective gaslighting” (see Sect. “Unjust interpretation and affective distortion”) when patients’ feelings are systematically *misinterpreted* in ways that make them doubt themselves. It can also, however, constitute the kind of *affective intimidation* at issue here, when patients who are perceived as “very, very troublesome” and “querulous” (Åsbring and Närvänen 2003, p. 715) or as “time-wasters” (Stenhoff et al. 2015, p. 202) are pressured into self-doubt to discourage them from further contesting diagnoses, demanding investigation, or insisting on accommodation and support. Indeed, many patients report avoiding consultations, hesitating to mention ME/CFS, and at times withdrawing from healthcare encounters altogether after repeated experiences of disbelief and dismissal (e.g., Melby and das Nair 2024).

### Unjust interpretation and affective distortion

Even when individuals are free from both indirect interference through unjust expectations and direct interference through unjust elicitation, affective injustice can arise at the level of *interpretation*. While there can always be misunderstandings, *unjust interpretations* arise when structural power imbalances systematically *distort* how the affective lives of marginalized individuals are understood. Two forms of such *affective distortion* are *affective pathologizing* (e.g., Eickers 2023) and *affective gaslighting* (e.g., Oliver 2025). *Affective pathologizing* occurs when valid affective reactions are interpreted as pathological symptoms of an underlying (psychological) disorder, as when a racialized person’s feeling of unease in a hostile environment is treated as paranoia. *Affective gaslighting* is a form of psychological manipulation in which members of dominant groups systematically invalidate the feelings of marginalized individuals, leading them to doubt their aptness or intelligibility, and sometimes even their own sanity, as when a woman’s anger at sexist slights is repeatedly dismissed as mere oversensitivity until she herself begins to question whether it is warranted (e.g., Whitney 2023). In both cases, the systematic misinterpretation *by others* distorts an individual’s affective life. But affective distortion can also occur when structural inequalities deprive marginalized individuals of the interpretive resources needed to make sense *of themselves*, giving rise to *affective inarticulation* (e.g., Pismenny et al. 2024). *Affective inarticulation* can be understood as a form of affective hermeneutical injustice (see Sect. “ME/CFS and epistemic injustice”) that arises when one is deprived of the resources to understand oneself, as when those subjected to repeated microaggressions feel persistent unease or anger, but are unable to make sense of these feelings because the dominant narratives that shape their sense of what they ought to feel suggest that nothing is wrong.

*Affective pathologizing* is well-documented in the context of ME/CFS. The biopsychosocial framing that still shapes public and medical understanding often distorts patients’ feelings through a psychiatric lens (e.g., Froehlich et al. 2022). In one study, as many as 90% of patients reported having been told at least once by health professionals that their symptoms were psychosomatic (Tschopp et al. 2023). As a result, legitimate feelings such as grief over lost capacities, anger at sometimes years of neglect, fear of deterioration, and frustration at dismissal are professionally recoded as signs of anxiety, depression, catastrophizing, or dysfunctional illness beliefs. The problem here is not primarily that healthcare practitioners assume, contrary to better evidence, that ME/CFS is a “mental illness,” but more fundamentally the prevailing social and scientific climate in which mental conditions are still treated as inferior. This hierarchical structure fuels the perception that they are “less real,” encouraging the view that patients’ experiences are “imagined, feigned, or exaggerated” (Spencer et al. 2025, p. 10) and markers of personal weakness or a “self-inflicted” psychological defect (Vink and Vink-Niese 2026). Pushing patients to understand themselves in such pathologizing terms not only impairs their ability to articulate their authentic experiences, but also discourages them from interpreting their affective lives in more positive terms, paving the way for *affective gaslighting*.

As already indicated, marginalized individuals experience *affective gaslighting* when dominant others interpret their experiences in ways that lead them to question their aptness and, in some cases, even their own grasp of reality. If they are internalized, the pathologizing medical framings just discussed can have precisely this effect. When healthcare practitioners repeatedly dismiss patients’ concerns out of ignorance, implicit bias, or medical paternalism and insist with the social authority of the white coat that they do “not have a real physical illness” and that it is “all in their head” (Bested and Marshall 2015, p. 224), patients may gradually come to lose confidence in what they themselves feel and mistrust their own, potentially more accurate, judgment (e.g., Au et al. 2022; Thoma et al. 2024). When this happens, medical dismissal not only undermines patients’ epistemic standing, but also their fundamental affective orientation, and becomes a form of affective gaslighting that can cause anxiety, depression, and, in severe cases, a withdrawal from social life. Affective gaslighting is, regrettably, a recurring theme in the ME/CFS literature (e.g., Smyth and Blitshteyn 2025; Thoma et al. 2024). Given that ME/CFS is an “invisible” condition, it is particularly vulnerable to dismissal. As a consequence, countless patients report “[g]aslighting, gaslighting, and more gaslighting” accompanied by “[l]iteral laughing from the medical providers” (Au et al. 2022, p. 6). Again, experiences of affective gaslighting can plausibly be intensified by gendered marginalization (e.g., Freeman and Stewart 2025; Lian 2020). Medical students are still taught, both implicitly and explicitly, that “women are unreliable reporters of their own symptoms and are more likely to have psychological rather than ‘real,’ physical diseases” (Plaza 2020, p. 102). As a consequence, women’s symptoms are more readily psychologized (Samulowitz et al. 2018), rendering them disproportionately vulnerable to gaslighting (e.g., Stosic et al. 2024). Importantly, when patients’ own grasp of what they are experiencing is systematically displaced by powerful medical “authorities,” this is not simply a matter of clinical misunderstanding, but a socially patterned distortion of their affective identity. As a consequence, while gaslighting begins in others’ misinterpretation, it can, as said above, culminate in patients *themselves* being unable to make sense of their affective lives, leading to *affective inarticulation*.

Constantly being disbelieved can result in an *affective inarticulation* that leaves ME/CFS patients with feelings of “emotional isolation […] and intense confusion” that makes them unable “to describe their condition to themselves or others” (Fennell et al. 2021, p. 4). Patients report having come to the conclusion that they must have “lost their mind” (Barnes 2023, p. 2), that they can no longer “trust themselves” (ibid.), that they “don’t really understand what [they] have […] turned into” (Fisher and Crawley 2013, p. 564), and even that they begin to seriously wonder whether they are “just using this as an excuse because [they] don’t like [their] life” (Dickson et al. 2008, p. 466). Additionally, there is general evidence that individuals with ME/CFS indeed experience reduced emotional awareness and difficulty identifying their feelings (e.g., Bileviciute-Ljungar and Friberg 2020; Maroti et al. 2017).

### Unjust responses and affective dismissal

Even when one’s feelings are correctly interpreted, one can still be affectively wronged if others *respond* in ways that refuse to acknowledge their significance. *Unjust responses* occur, for instance, when feelings of marginalized individuals are disregarded, trivialized, or belittled, not on substantive grounds, and not merely because someone is being generally rude or insensitive, but simply because they are “this kind of people.” Two important forms of such *affective dismissal* are *affective invalidation* (e.g., Pismenny et al. 2024) and *affective silencing* (e.g., Bogaert 2025). *Affective invalidation* occurs when someone’s feelings are dismissed as unfounded or exaggerated or otherwise denied the significance they deserve, as when a trans person’s distress at being misgendered is brushed aside as an overreaction. *Affective silencing* can be understood as an extreme form of affective dismissal which involves active suppression of others’ feelings or of their uptake, as when women in abusive relationships are threatened with violence if they disclose their fear and distress, thereby effectively “gagging” those experiences before they can receive public uptake.

Experiences of *affective invalidation* are, again, widely reported in the ME/CFS literature (e.g., Kendrick and Beesley 2016). Some instances are rooted in affective stereotyping (see Sect. “Unjust expectations and affective policing”), as when potentially apt anger and frustration at neglect are dismissed as aggression or hostility under the stigma of the “militant” ME/CFS patient (Blease and Geraghty 2018), or when fear of deterioration and grief over lost capacities are cast as signs of psychological instability in light of the “hysterical woman” stereotype (e.g., Hsu 2024). Other instances take the form of affective pathologizing (see Sect. “Unjust interpretation and affective distortion”), as when experiences are trivialized through the distorting lens of ME/CFS as a “mental illness,” or arise in contexts of affective gaslighting (see Sect. “Unjust interpretation and affective distortion”), where experiences are denied significance and recast as figments of patients’ imagination (Muirhead et al. 2021, p. 2). But not all affective invalidation takes such dramatic forms. ME/CFS patients also encounter more ordinary forms of dismissal which are often no less frustrating, for instance when family members or friends, often with good intentions, respond to severe suffering with comments or advice that minimize its seriousness. This happens, for instance, when individuals with ME/CFS are asked whether they “have […] tried taking vitamin B12” (Waite and Elliot 2021, p. 164), told that “[m]any people have it worse” (Smyth and Blitshteyn 2025, p. 6), assured that their symptoms are merely “due to stress” (Sirotiak 2023, p. 1257) or to the fact that they “just notice symptoms more” (ibid.), or consoled with remarks such as “At least it’s not cancer” (Smyth and Blitshteyn 2025, p. 6). While such remarks may seem minor innocent blunders or insensitivities when taken in isolation, they amount to an affective injustice when they are socially patterned, structurally enabled, and endlessly reproduced by the lack of any broader pressure on the majority to understand ME/CFS well enough to avoid them.

Affective invalidation can shade into *affective silencing*, especially when it reaches a broader audience in public contexts. For instance, the stereotype of the “militant” ME/CFS patient (Blease and Geraghty 2018) does not merely trivialize individual experiences; it also works, at a collective level, to neutralize and silence the affective force of patients’ anger and outrage. Affective silencing is also effected by public discourse that ridicules ME/CFS. The slur “Yuppie Flu” (see Sect. “ME/CFS: history, models, and burdens”), for instance, not merely trivializes ME/CFS; it frames those who live with it as pampered complainers whose distress is exaggerated, inviting lay audiences to regard their experiences not as an intelligible response to suffering and neglect, but as yet more evidence that “people like them” are making a fuss. A particularly egregious form of affective silencing occurred in the controversy surrounding the PACE trial. There, reports by ME/CFS researchers that they had experienced harassment and death threats circulated within a broader discursive framing of ME/CFS patients and critics as “militant,” “fundamentalist,” violent, or irrational (e.g., Blease and Geraghty 2018; Hunt 2025). In a subsequent freedom-of-information appeal, a tribunal described one assessment of “activist behaviour” as “grossly exaggerated” (Lubet 2017, p. 1203) and testimony stated that “no threats have been made either to researchers or participants” (Agardy 2017, p. 1207). But regardless of the accuracy of individual reports or the motives of those involved, framing patients and critics through the figure of the dangerous or irrational activist shifted attention away from the substance of their concerns and toward their alleged character, silencing their anger and calls for support before they could receive serious uptake.

### Unjust benefits and affective exploitation

Managing one’s affective life can be demanding, both mentally and physically. When socially dominant groups derive *unjust benefits* from the “emotional labor” (Hochschild 1983) that marginalized individuals must invest to navigate structural power imbalances, for instance when women are expected to endure sexist treatment while remaining calm, agreeable, and emotionally accommodating for men’s comfort, this constitutes a form of *affective exploitation* (Whitney 2018). Two important manifestations are *affective double burdening* (e.g., Pismenny et al. 2024) and *affective appropriation* (e.g., Agathangelou 2019). *Affective double burdening* occurs when those already suffering injustices are additionally also expected to manage the needs of others, often of those implicated in the original injustice, as when marginalized individuals are expected not only to bear the effects of discrimination, but also to reassure those responsible that they have done nothing seriously wrong. *Affective appropriation* occurs when the affective lives of marginalized groups are exploited by dominant actors or institutions for ideological or commercial ends, as when corporations exploit feminist or anti-racist rhetoric to market their brand while leaving untouched the conditions of inequality that make such rhetoric resonant in the first place.

One double burden experienced by individuals with ME/CFS consists in living with severe symptoms while also having to make their condition intelligible to others who are unable, or unwilling, to understand it, for instance by providing physicians with the scientific evidence needed for it to be recognized as legitimate. Since this requires energy that they do not have, it can further exacerbate their condition. This predicament has a distinctly *affective* dimension insofar as individuals with ME/CFS are often required to render their own suffering affectively manageable for others. Stigma from family members, friends, employers, and clinicians can compound the distress associated with the illness by forcing patients to explain themselves, absorb blame, and manage others’ reactions while already exhausted and unwell (e.g., McManimen et al. 2018). This can mean having to endure “total disbelief and refusal to accept or understand [the] illness” from one’s own family (Murray and Turner 2023, p. 61), ranging from wearying questions such as “Why are you sleeping all the time? The doctor says there is nothing wrong” (Drachler et al. 2009, p. 10), to accusations that the symptoms are being “intentionally used for advantage” (Crix et al. 2012, p. 3), and even to the brutal declaration by one’s parents that one is “no longer their daughter” (Murray and Turner 2023, p. 61). In a supportive family environment, this double burden takes a different form, but is often no less taxing. Here, it can involve the additional emotional work of protecting significant others from worry or strain by carefully managing how one presents one’s illness. This may include silencing or otherwise hiding one’s own feelings of guilt about causing trouble or being unable to “be social or hav[e] a sexual relationship” (Stormorken et al. 2017, p. 8), as well as one’s feelings of shame about not being able to fulfil the role one takes oneself to be expected to fulfil (Williams et al. 2019, p. 268). It can also involve caring for the feelings of loved ones, for instance by reassuring them that one is not “giving up” or “losing hope” (e.g., Brittain et al. 2021). A related form of double burdening arises from the need to manage how one’s suffering appears to the broader public that requires packaging one’s feelings of fear, anger, and despair in ways that remain credible without appearing threatening, so that one is visible enough to be recognized, yet not so visible that one appears “exaggerated.”

Popular media have the power to increase visibility and challenge prejudices among a broad audience by featuring characters explicitly portrayed as having ME/CFS. In practice, however, they often exploit the condition for entertainment and commercial gain, while reinforcing the very clichés that sustain its stigmatization. This can be understood as a form of *affective appropriation*. Just as the exploitation of the Jim Crow caricature helped marginalize Black people by turning racist stereotypes of laziness, subservience, and naïveté into entertainment for white audiences, stereotypes about ME/CFS can be used to generate comic relief, casting patients as oversensitive hypochondriacs who are unable to distinguish ordinary tiredness from illness or as malingerers who feign illness to avoid responsibility. In 1989, the fifth season of *The Golden Girls* began with the two-part episode “Sick and Tired,” in which Dorothy seeks help for severe fatigue but is repeatedly told by doctors that it is just a consequence of age, femininity, nervous strain, or personal disposition, until she is finally diagnosed with CFS. The episode has rightly been praised for making CFS visible and exposing medical gaslighting. But it has also been criticized for containing that suffering within the disposable logic of the sitcom, not least because Dorothy’s health is effectively “reset in subsequent episodes” (Franklin 2025, p. 78). Since a series built around largely self-contained episodes leaves little room to represent the chronic and fluctuating reality of an incurable illness, it was effectively reduced to a one-off narrative that did not address the difficulties of living with it, but focused on the struggle to secure diagnosis and vindication.

A more troubling example is the 2004 pilot of the drama *House M.D.* The episode introduces House as the brilliant, but unconventional and cynical diagnostician, when he immediately casts a patient who believes he has CFS or fibromyalgia as a hypochondriac. Ultimately, the episode vindicates that judgment when House “cures” the patient with placebo mints in a Vicodin bottle, reinforcing the suspicion that the illness is psychosomatic. Most notably, Netflix’s 2018 series *Afflicted*, initially marketed as a compassionate documentary, drew intense criticism after participants and an open letter signed by physicians and scientists accused it of framing ME/CFS as exclusively psychosomatic, editing interviews to heighten skepticism, foregrounding psychological speculation from clinicians who had never examined the participants, and omitting biomedical evidence that might have supported a non-psychiatric understanding of their illnesses (Nilsson 2025). Eventually, seven participants sued Netflix, complaining that they had been lured into a salacious reality-TV narrative that portrayed them as “lazy, crazy, hypochondriacs and/or malingerers who were deserving of scorn” (Harrison 2019).

Another important form of affective appropriation of ME/CFS is the commodification of hope. In a context of diagnostic uncertainty, limited treatment options, and repeated experiences of disbelief, a market emerges not merely for advice or support, but for the hope for recovery itself. This creates a space for “illness entrepreneurs” (Geiger and Loza 2026) to offer one-to-one sessions and group programs that commodify the promise of an escape from a painful present. This exploits patients’ desperation and vulnerability for profit without addressing the underlying structural deficits. Lastly, a 2022 study analyzed 452 images used in Google News coverage of ME/CFS and 3,400 ME/CFS-related images drawn from stock-image platforms (Hallmann et al. 2022). It found that only 13.5% of the news images were authentic depictions of reality, whereas the rest—and *all* of the stock images—recycled familiar tropes, such as an office-dressed woman with a hand to the head, propagating a picture of ME/CFS as ordinary tiredness and minimal disability. This is not just blatant stereotyping (see Sect. “Unjust expectations and affective policing”), but a form of exploitation: what is commercially useful to media outlets and image platforms is not truthful representation, but a simplified and marketable depiction that aims at consumption rather than understanding, once again reinforcing stigma.

If the analysis in this section is on the right track, individuals with ME/CFS are subject to distinct forms of affective injustice structurally similar to those affecting other marginalized groups (see Table 1). The struggle over ME/CFS is therefore not only a struggle over evidence, classification, causation, and models, but also over whose suffering is socially allowed to appear as real, whose affective responses are granted legitimacy, and the right to be ill without having to dress up one’s illness in compliant or commercially useful ways. To describe this additional layer of harm as a decidedly “affective” injustice is not to deny the importance of other factors, but to insist that persistent disbelief can damage more than patients’ status as “knowers.” The same social and institutional structures that discredit them epistemically can also damage their affective autonomy and authenticity, their affective attunement to others, and their capacity for affective sense-making and agency. Accordingly, what we need is not just better science, but also more just social and institutional structures. I conclude by addressing some objections and drawing out some broader implications.

## Conclusion: objections and implications

A first objection might be that the phenomena discussed in Sect. “ME/CFS and affective injustice” can already be captured by the notion of epistemic injustice. If patients are invalidated, stereotyped, or pressured into adapting how they communicate their illness, one might say, they are, at root, wronged as knowers, and their affective suffering is just a downstream psychological consequence that might be lamentable, but does not add anything distinctive. I do not think this is right. Epistemic and affective injustices can go hand in hand, but they do not coincide. Even if patients were respected “as knowers,” they could still suffer under conditions of structural imbalance in ways that, in light of the considerations in Sect. “From epistemic to affective injustice”, are genuinely unjust in their own right. The notion of affective injustice thus brings into view dimensions of marginalization that a focus on testimony and understanding alone misses. It thereby helps explain why acknowledging and avoiding epistemic wrongs is important, but not enough.

A second concern is that the notion of affective injustice may unduly moralize ordinary interpersonal failures or legitimate clinical uncertainty. Of course, not every insensitive response, diagnostic hesitation, or scrutiny of a patient’s account constitutes an injustice. What matters is whether socially entrenched practices and power asymmetries systematically expose marginalized individuals to affective harm. Clinicians may acknowledge genuine uncertainty without affectively wronging patients, if such caution is expressed with due humility and does not slide into humiliation, stereotyping, or the pathologization of fitting affective responses.

Another objection might be that the foregoing considerations boil down to little more than a general complaint about suffering under chronic illness. There is some truth in this. I have not meant to claim that individuals with ME/CFS are affectively harmed in wholly unique ways. But the contested status of ME/CFS and its history of psychologization and gendered trivialization make affective injustices especially vivid, rendering ME/CFS a paradigmatic case for understanding how social and institutional structures can shape the affective lives of marginalized individuals in unjust ways. At the same time, this is not to deny that similar forms of affective injustice can, and arguably do, harm patients with other contested illnesses as well. But this makes things worse, not better.

Lastly, one might worry that a focus on affective injustice diverts attention from more urgent concerns. What individuals with ME/CFS need, one might say, is biomedical research, accurate diagnosis, disability support, and income security, not “woke” ruminations about what is at bottom a political and institutional problem. I think the opposite is the case. Foregrounding affective injustice does not mean watering down the “hard realities” of medicine, healthcare, and insurance or social security systems with a concern for “subjective sensitivities,” but making visible how those realities must in fact be lived and endured. The affective harms discussed above are not detachable from material deprivation, clinical neglect, or welfare exclusion, but part and parcel of the mechanisms through which those realities are enacted and reproduced. Affectivity is not an optional anecdotal “add-on” to allegedly objective categories (e.g., Carel 2016; Shai et al. 2021), but part of what illness as a socially situated human reality *is*. To be affectively invalidated, gaslit, silenced, or exploited is not merely to feel even more bad in an already difficult situation, but to be systematically positioned by social and medical institutions in ways that can worsen illness, obstruct access to care, and undermine one’s practical agency. Making this explicit makes the more material concerns even more pressing.

If the considerations in this paper are by and large along the right lines, they have practical implications. Improving care for people with ME/CFS requires more than better diagnostics and treatment: clinicians and institutions must also recognize that anger, grief, fear, mistrust, or exhaustion may be fitting responses to illness and prolonged disbelief rather than signs of poor coping or therapeutic resistance. Clinical environments should not reward performances of optimism and compliance while penalizing those who cannot or will not sustain them. This requires education about the social history of ME/CFS, space for uncertainty without humiliation, and policies that do not make care or support conditional upon affective performances of deservingness.

Lastly, the considerations in this paper suggest that the philosophy of medicine must attend not only to the metaphysical nature of a medical condition and the epistemic status of our best medical knowledge (e.g., Reiss and Ankeny 2025), but also to the ways in which medical practice normatively shapes patients’ affective lives. Illness is not only classified, explained, and treated, but also felt and socially managed, typically under conditions of unequal power. We thus need concepts that capture not only failures of knowledge, but also failures of affective uptake, distortions of emotional meaning, and the exploitation of vulnerability. Affective injustice is one such concept.

With all that being said, philosophy should not have the last word. If the notion of affective injustice has any value, it lies, ultimately, in helping us hear more clearly what marginalized individuals have to say. The last word is therefore owed to those whose illness has too often been doubted, psychologized, trivialized, or made to fit the needs of others. What they want is neither pity nor theoretical sophistication, but—apart from having their health back—justice: the chance to be ill without disbelief, without humiliation and stigmatization, and without having to translate their suffering into affective performances dominant others find acceptable or useful.

## Footnotes

1 Specifically, I highlight eight such aims (Walter 2026). *Descriptively*, a notion of affective injustice should (1) bring otherwise disparate affective harms under a common framework, (2) articulate what makes them distinctively affective, and (3) furnish the hermeneutical resources needed to recognize and interpret them as socially patterned wrongs. *Normatively*, it should (4) challenge entrenched norms, (5) locate responsibility, and (6) specify positive ideals of affective justice. *Pragmatically*, it should (7) motivate institutional and social change and (8) foster self-understanding and self-respect among those whose affective lives are unjustly impaired.

2 An earlier version of this taxonomy was formulated in Walter and aus dem Bruch (2025), based on prior pioneering work by Pismenny et al. (2024). Here, it is amended and enriched.

3 While existing qualitative studies amply document disbelief, stigmatization, emotional distress, altered self-presentation, and coercive healthcare encounters among individuals with ME/CFS, they were not designed specifically to investigate affective injustice. I therefore draw on the patient accounts reported in these studies to illustrate experiences that may be interpreted through the lens of affective injustice, without suggesting that this alone establishes the prevalence, empirical discreteness, or exhaustive coverage of the proposed categories. A separate empirical study currently underway examines more systematically how individuals with ME/CFS themselves describe, evaluate, and differentiate these forms of affective harm (Walter forthcoming).
